# Effect of Intraoperative Dexmedetomidine Dose on Postoperative First Night Sleep Quality in Elderly Surgery Patients: A Retrospective Study With Propensity Score-Matched Analysis

**DOI:** 10.3389/fmed.2020.00528

**Published:** 2020-10-06

**Authors:** Jingjing Cai, Yuanjing Chen, Xuechao Hao, Xiwen Zhu, Yaxing Tang, Sheng Wang, Tao Zhu

**Affiliations:** ^1^Department of Anesthesiology and Translational Neuroscience Center, West China Hospital, Sichuan University & The Research Unit of West China (2018RU012), Chinese Academy of Medical Science, Chengdu, China; ^2^Department of Anesthesiology, The Second Affiliated Hospital, Chongqing Medical University, Chongqing, China; ^3^Department of Anesthesiology, Guangdong Cardiovascular Institute, Guangdong General Hospital, Guangdong Academy of Medical Sciences, Guangzhou, China

**Keywords:** dexmedetomidine, surgery, dose, sleep, elderly patients

## Abstract

**Background:** Postoperative sleep disorder is common in elderly surgery patients, and it often worsens their recovery after surgery. This study aimed to explore the effect of intraoperative dexmedetomidine dose on postoperative sleep quality.

**Methods:** Based on information regarding dexmedetomidine use during surgery from an electronic medical record system, 4,349 elderly surgery patients were divided into three groups: 1,374 without intraoperative use of dexmedetomidine (Non-DEX), 917 with dexmedetomidine 0.1–0.2 μg/kg/h (Low-DEX), and 2,058 with dexmedetomidine >0.2 μg/kg/h (High-DEX). The numerical rating scale (NRS) for sleep disturbance during the first night after surgery was recorded, and the incidence of NRS ≥ 6 was considered the primary outcome.

**Results:** NRS (*P* < 0.001) and incidence of severe sleep disturbance (*P* < 0.001) were lower in patients receiving intraoperative dexmedetomidine than in those without the intraoperative use of dexmedetomidine. Patients in the Low-DEX group had the lowest incidence, followed by those in the High-DEX and Non-DEX groups (6.7% vs. 13.7% vs. 19.5%). After propensity score matching, 906 pairs of elderly surgery patients were included in the Low-DEX and High-DEX groups, and the Low-DEX group had lower NRS (2.7 ± 2.1 vs. 3.1 ± 2.4, *P* < 0.001) than the High-DEX group. The incidence of severe sleep disturbance was lower in the Low-DEX group than in the High-DEX group (6.6% vs. 12.8%) with an odds rate of 0.48 (95% confidence interval, 0.35 to 0.67).

**Conclusions:** For elderly patients, intraoperative dexmedetomidine use can significantly improve the quality of the first night sleep after surgery. Low-dose (0.1–0.2 μg/kg/h) dexmedetomidine can have an improvement effect on sleep quality, and it is recommended to improve the quality of postoperative sleep.

## Introduction

For elderly patients undergoing surgery, postoperative sleep disorder is a very common clinical problem that may worsen the patients' recovery after surgery, e.g., increasing the risk of inadequate postoperative pain control, delirium, or cognitive dysfunction (1–5). In addition, one study has demonstrated that enhancing sleep quality after total shoulder arthroplasty can directly improve the patients' functional outcomes (6). Numerous methods have been used to improve sleep quality after surgery, such as using blinders and earplugs to eliminate noise and light and using melatonin and sedative-hypnotic drugs (7–10). However, few studies have currently attempted to improve postoperative sleep quality during the intraoperative period.

Two previous studies found that dexmedetomidine, an α2 adrenoreceptor agonist, has the potential to improve the sleep quality of elderly postoperative patients (11, 12). Continuous infusions of low-dose dexmedetomidine (0.1 μg/kg/h) at night can improve patients' postoperative first night sleep quality in the intensive care unit (ICU) by changing the patients' sleep structure (12). Furthermore, one randomized small sample size study demonstrated that intraoperative continuous infusions of dexmedetomidine (0.1 μg/kg/h) can improve the first night sleep quality in elderly patients following lung cancer surgery (13). Another recent randomized study found that dexmedetomidine use (0.2–0.7 μg/kg/h) during a daytime operation can better improve postoperative sleep quality in patients (aged 30 to 55) undergoing laparoscopic abdominal surgeries (14). These studies demonstrated that intraoperative dexmedetomidine use may be an optional strategy to improve the postoperative sleep quality of elderly surgery patients. However, these studies were restricted to several specific populations and established study protocol, and the dexmedetomidine dose that is optimal to improve postoperative sleep quality for elderly surgery patients remains unknown. Therefore, this study aimed to retrospectively recruit elderly surgery patients to explore the effect of intraoperative dexmedetomidine dose on postoperative sleep quality.

## Methods

### Patients

**Figure 1:**
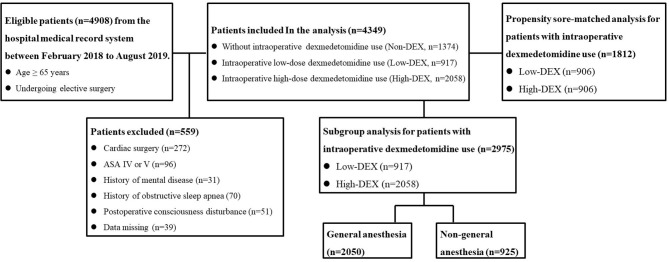
The study population with the inclusion and exclusion criteria.

This study was conducted according to the STROBE guidelines as a single-center, retrospective observational study (15). All procedures performed in studies involving human participants were in accordance with the ethical standards of the institutional and/or national research committee and with the 1964 Helsinki Declaration and its later amendments or comparable ethical standards. The study protocol was approved by the Institutional Review Boards of the Second Affiliated Hospital, Chongqing Medical University. Since the current study was designed as a retrospective study based on the hospital medical record system, informed consent from the patients was waived. All unidentifiable patient data for analysis in the study can be acquired from the corresponding author TZ through email. As shown in Figure 1, a total of 4,908 elderly patients (age ≥ 65 years) undergoing elective surgery were inquired and collected from the hospital electronic medical record system from February 2018 to August 2019. The exclusion criteria included cardiac surgery, American Society of Anesthesiologists Physical Status Classification (ASA class) IV or V, history of mental disease, obstructive sleep apnea, coma, or unconsciousness.

### Baseline Data and Surgical Information of Intraoperative Interventions

Based on the hospital electronic medical record system, basal demographic data such as sex, age, height, weight, BMI, and smoking status were collected. The ASA class and common diseases of the elderly, including hypertension, cardiac disease, diabetes, and pulmonary disease, were recorded. According to the surgical site and types, patients were categorized as abdominal operation, orthopedic operation, neurological operation, or other operation. According to whether the time of operation was ≥2 h or not, the surgeries were divided into major or non-major surgeries, and the surgery methods were classified as minimally invasive and non-minimally invasive surgery. In addition, because one study reported that dexmedetomidine use during daytime and nighttime surgery has different effects on sleep quality (14), we also collected the starting times of the surgeries for all the included patients. The starting times were classified as morning (0:00–12:00 AM) and afternoon (12:00–24:00 PM).

### Anesthetic and Analgesia Strategies

In the hospital, anesthetic strategies were selected according to the anesthetists' comprehensive evaluation based on the types of surgery, preoperative complication, patients' wishes, and operating room environment and conditions. In this study, the anesthesia methods recorded were grouped into general anesthesia or non-general anesthesia. For some major surgeries, including thoracic surgery, abdominal surgery, and neurosurgery, general anesthesia was often performed, while for orthopedic and urinary surgeries, non-general anesthesia was often performed. Whether or not dexmedetomidine was used during the surgery and the mean dosage of dexmedetomidine according to patients' weight and time of operation were also recorded. In the hospital, dexmedetomidine usage and its dosage was determined by the anesthesiologist according to his/her experience and the patients' status. However, dexmedetomidine was administered via a pump at 0.1 to 0.6 μg/kg/h after anesthesia induction for general anesthesia patients and after the spinal anesthesia or nerve block for non-general anesthesia patients. In addition, for the primary analysis of the current study, patients were grouped as low-dose (Low-DEX, 0.1–0.2 μg/kg/h, ≤ 1/3 of the highest dose in the current population) and high-dose (High-DEX, >0.2 μg/kg/h) populations according to the dexmedetomidine dose. Furthermore, whether patients receiving postoperative patient-controlled intravenous analgesia (PCIA) with opioids provided by the acute pain service group was also recorded. In the hospital, sufentanil and flurbiprofen axetil were routinely combined and applied in PCIA, of which parameters were adjusted according to surgery type and patients' personal details.

### Postoperative Outcomes

From February 2018 onward, a specialized postoperative visiting team was established for surgery patients' follow-up in the hospital. All surgery patients were interviewed at 12, 24, and 48 h after the surgery. The postoperative visiting team was independent of the anesthetists' work in the surgical room and they were blind to the patients' intraoperative intervention. The investigators were asked to record the surgery patients' subjective first night sleep quality using the numerical rating scale (NRS, 0 to 10, which “0” represents excellently good sleep and “10” represents could not completely fall asleep during the whole night) (11, 16, 17). In the study, the primary outcome was set as the incidence of severe sleep disturbance (SSD), which was defined as NRS ≥6, indicating that sleep was repeatedly interrupted during the whole night or even worse.

Beyond that, the postoperative visiting doctors also routinely assessed the patients' pain intensity (NRS, “0” represents no pain, while “10” represents intolerable pain) at 24 and 48 h after surgery. If a patient presented with severe pain (NRS ≥ 4) and required additional analgesia intervention at 48 h after surgery, the patient was recorded as case requiring additional analgesia (18, 19). In addition, whether the patient can independently perform off-bed activities at 48 h after surgery and hospital stay for all the surgery patients were also recorded. Postoperative complications, including bradycardia and hypotension, during 48 h after the surgery that may develop because of dexmedetomidine were recorded. Bradycardia was defined as heart rate <55 bpm or a decrease of >20% from baseline and hypotension as systolic blood pressure <95 mmHg or a decrease of >20% from baseline (11).

### Power Calculation

In the current study, the incidence of SSD were considered the primary endpoint, and propensity score-matched (PSM) analysis was considered the primary analysis. PASS software version 11.0 (NCSS, Hayesville, UT, USA) was used to calculate the power according to a two-sided group comparison and a sample size of 906 in the PSM analysis achieved a power of 0.994 to detect a rate difference of 6.2% in incidence of SSD between the Low-DEX and High-DEX groups.

### Statistical Analysis

In the study, all variables were assessed and analyzed using the R statistical software, and a two-sided *P* < 0.05 was considered statistically significant. Continuous variables are presented as mean ± standard deviation, and categorical variables are presented as number (percentage). Firstly, patients were grouped as Low-DEX, High-DEX, and Non-DEX (without intraoperative dexmedetomidine use) populations. Continuous baseline and outcome data between the three groups were compared using one-way analysis of variance or a Kruskal-Wallis test and *post-hoc* multiple comparisons were performed using Bonferroni corrections. The *x*^2^ test or Fisher's exact test was used to compare categorical variables among the three groups, and Bonferroni corrections were also used to perform *post-hoc* multiple comparisons.

Secondly, an enter model logistic regression analysis was performed to identify the potential effect factors for SSD after the surgery. The baseline variables including sex, BMI group (≤ 18.5 kg/m^2^, 18.5–25 kg/m^2^, ≥25 kg/m^2^), smoking (yes/no), ASA class, surgery duration (<2 h or ≥2 h), surgery types and time periods (morning or afternoon), minimally invasive surgery (yes/no), general anesthesia (yes/no), with or without hypertension, cardiac disease, diabetes, and pulmonary disease, and whether or not receiving PCIA were included in the model. Then, based on the baseline factors (*P* < 0.05) with differences between patients of the Low-DEX and High-DEX groups and the identified factors (*P* < 0.05) in the logistic model, we performed a PSM analysis to further compare the effect of intraoperative dexmedetomidine dose on postoperative sleep quality. Matching using the 1:1 nearest neighbor method without replacement under a logit model was performed. After matching analysis, Student's *t*-tests, Mann–Whitney tests, and *x*^2^ tests were performed to compare the difference between the two groups according to the types of variables. In addition, the relative risk with 95% confidence interval (CI) was calculated for the primary outcome.

Thirdly, subgroup analysis including all patients who received dexmedetomidine during the surgery was performed according to the two anesthesia methods that the patients received. The presence or absence of severe postoperative SSD was considered as the dependent outcome. Step-wise logistic regression analysis was performed and all the baseline variables and dexmedetomidine doses (Low-DEX or High-DEX) were included in the model. An odds rate with 95% CI was, respectively, calculated for the two populations.

## Results

**Figure 2:**
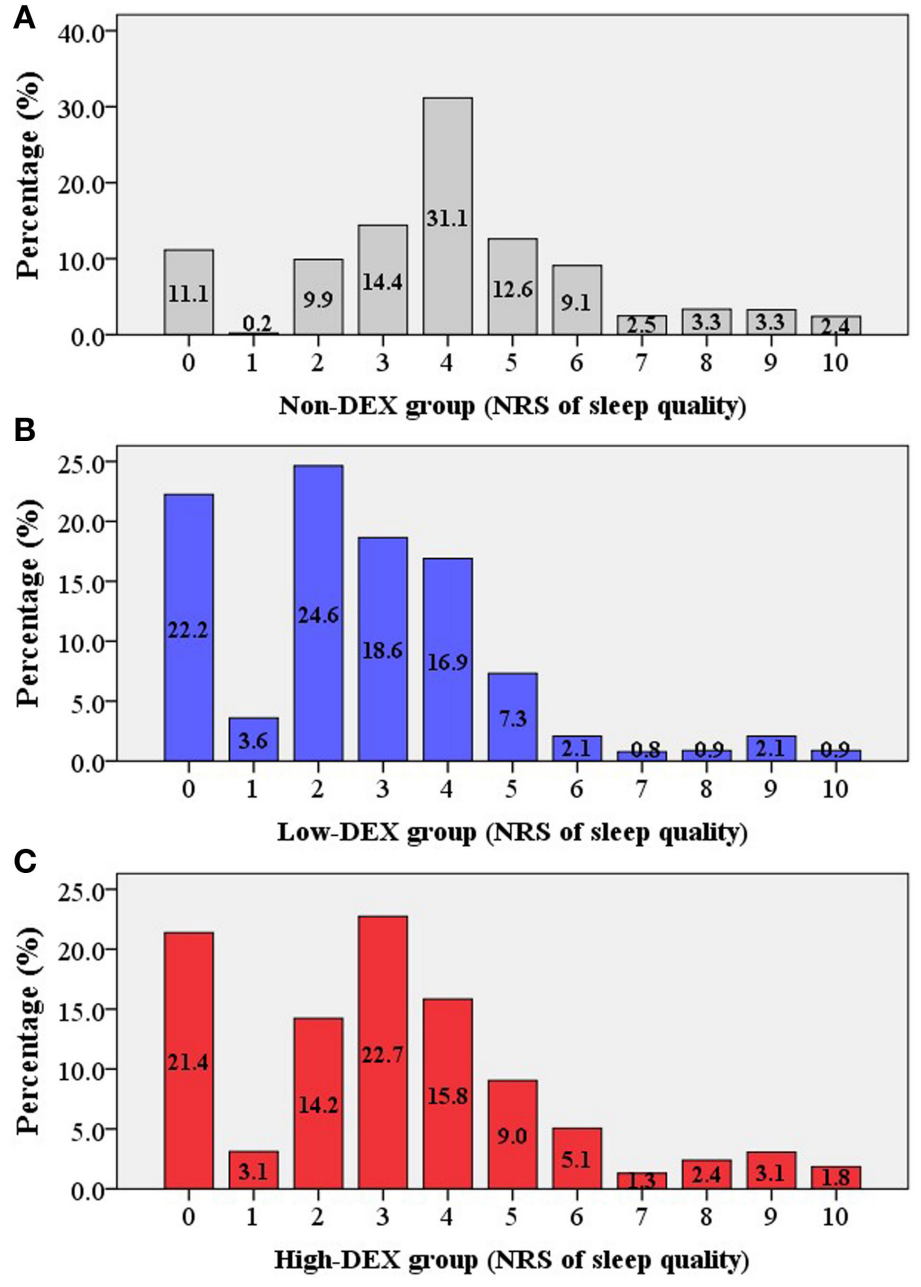
Bar-chart plot of the NRS of sleep quality in the different groups [**(A)** Non-DEX group; **(B)** Low-DEX group; **(C)** High-DEX group]. Box, percentage.

As shown in Figure 1, a total of 4,908 elderly patients were inquired and 4,349 cases were included in the final analysis according to the inclusion and exclusion criteria. Demographic and preoperative data of all the included patients in the Non-DEX, Low-DEX, and High-DEX groups were listed in Table 1. Compared to patients who did not receive intraoperative dexmedetomidine, there were significant differences in age, BMI, rate of preoperative cardiac or pulmonary disease, surgery duration, rate of major surgery, surgery type, rate of general anesthesia, and receiving PCIA for patients receiving intraoperative dexmedetomidine. A difference was found in age, BMI, rates of pulmonary disease, and whether or not the patient underwent general anesthesia between the Low-DEX and High-DEX groups. The bar-chart plot of NRS of sleep quality by group (Non-DEX, Low-DEX, and High-DEX) is shown in Figure 2.

**Table 1:** Clinical characteristics at baseline and postoperative outcomes between the Non-DEX, Low-DEX, and High-DEX patient groups.

	**Non-DEX (*n* = 1,374)**	**Low-DEX (*n* = 917)**	**High-DEX (*n* = 2,058)**	***P*-value**
Age; year	72.2 ± 6.0	71.9 ± 5.7	70.9 ± 5.3^*^^#^	<0.001
Male; *n* (%)	715 (52.0%)	540 (58.9%)^*^	1173 (57.0%)^*^	0.002
BMI; kg/m^2^	24.1 ± 3.5	23.6 ± 3.5^*^	24.2 ± 3.5^#^	<0.001
ASA; *n* (%)				0.852
Class II	512 (37.3%)	337 (36.8%)	778 (37.8%)	
Class III	862 (62.7%)	580 (63.2%)	1280 (62.2%)	
Smoking status; *n* (%)	123 (9.0%)	107 (11.7%)	218 (10.6%)	0.093
Hypertension; *n* (%)	457 (33.3%)	344 (37.5%)	682 (33.1%)	0.049
Cardiac disease; *n* (%)	269 (19.6%)	143 (15.6%)^*^	336 (16.3%)^*^	0.016
Diabetes; *n* (%)	181 (13.2%)	126 (13.7%)	286 (13.9%)	0.828
Pulmonary disease; *n* (%)	336 (24.5%)	285 (31.1%)^*^	548 (26.6%)^#^	0.002
Operation in the morning; *n* (%)	631 (45.9%)	407 (44.4%)	969 (47.1%)	0.386
Surgery duration; h	2.0 ± 1.3	2.2 ± 1.4^*^	2.3 ± 1.4^#^	<0.001
Major surgery (≥2 h); *n* (%)	568 (41.3%)	455 (49.6%)^*^	1058 (51.4%)^*^	<0.001
Surgery type; *n* (%)		^*^	^*^	<0.001
Abdominal surgery	360 (26.2%)	177 (19.3%)	440 (21.4%)	
Urological surgery	259 (25.4%)	262 (28.6%)	499 (24.2%)	
Orthopedic surgery	521 (34.7%)	298 (32.5%)	681 (33.1%)	
Other type surgery	234 (27.5%)	180 (19.6%)	438 (21.3%)	
Minimally invasive surgery; *n* (%)	486 (35.4%)	317 (34.6%)	728 (35.4)	0.903
General anesthesia; *n* (%)	1025 (74.6%)	601 (65.5%)^*^	1449 (70.4%)^*^^#^	<0.001
Received PCIA; *n* (%)	461 (33.6%)	385 (42.0%)^*^	840 (40.8%)^*^	<0.001
Pain NRS at 24 h after surgery	1.2 ± 1.3	1.3 ± 1.3	1.3 ± 1.4	0.993
Pain NRS at 48 h after surgery	1.7 ± 1.2	1.5 ± 1.2	1.6 ± 1.1	0.288
Additional analgesia requirement during 48 h after surgery; *n* (%)	132 (9.6%)	66 (7.2%)	250 (12.1%)^#^	<0.001
Sleep quality NRS	4.0 ± 2.3	2.7 ± 2.1^*^	3.1 ± 2.4^*^^#^	<0.001
Severe sleep disturbance; *n* (%)	268 (19.5%)	61 (6.7%)^*^	281 (13.7%)^*^^#^	<0.001
Bradycardia; *n* (%)	216 (15.7%)	131 (14.3%)	427 (20.7%)^*^^#^	0.001
Hypotension; *n* (%)	354 (25.8%)	259 (28.2%)	650 (31.6%)^*^	<0.001
Independently off-bed activity at 48 h after surgery; *n* (%)	205 (14.9%)	154 (16.8%)	317 (15.4%)	0.465
Hospital stay; day	7.0 ± 2.5	6.0 ± 2.5^*^	6.7 ± 2.6^*^^#^	<0.001

*BMI, body max index; ASA, American society of anesthesiologists; NRS, number rating scale; PCIA, patient-controlled intravenous analgesia*.

* *P < 0.05, compared to Non-DEX group*;

# *P < 0.05, compared to Low-DEX group*.

**Figure 3:**
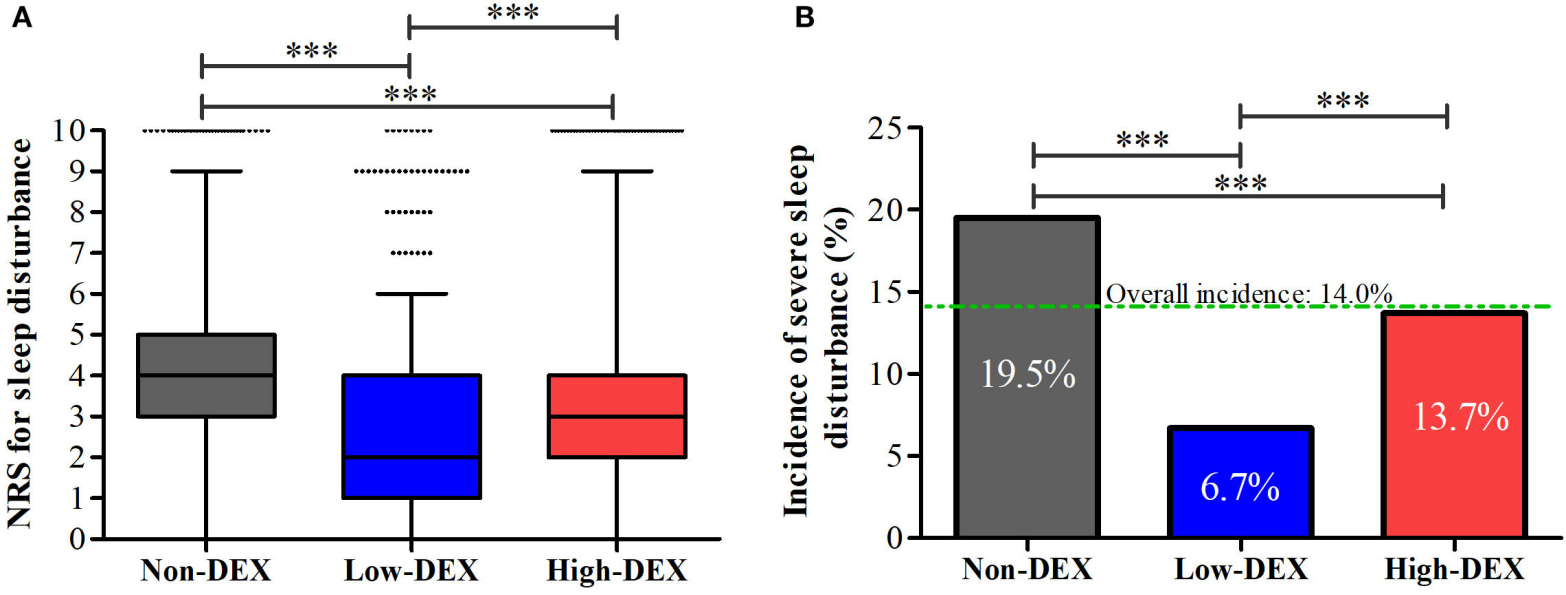
NRS for sleep disturbance **(A)** and the incidence of severe sleep disturbance **(B)** during the first night after the surgery in the Non-DEX group (*n* = 1,374), Low-DEX group (*n* = 917), and High-DEX group (*n* = 2,058) patients. ^***^P < 0.001; **(A)** box plot, interquartile range; solid line, median; whiskers, third quartile + 1.5^*^ interquartile range; dots, abnormal value. **(B)** box, percentage.

As shown in Figure 3A, distributions of NRS for sleep disturbance in different groups were presented; the distribution of NRS for sleep disturbance in the Low-DEX (2.7 ± 2.1 vs. 4.0 ± 2.3, *P* < 0.001) and High-DEX (3.1 ± 2.4 vs. 4.0 ± 2.3, *P* < 0.001) groups were significantly lower than that in the Non-DEX group, and it was lower in the Low-DEX group than in the High-DEX group (*P* < 0.001). Furthermore, significant differences regarding the incidence of SSD existed among the three groups, patients in the Low-DEX group had the lowest incidence and it was lower in the High-DEX group than in the Non-DEX group (6.7% vs. 13.7% vs. 19.5%, *P* < 0.001, Figure 3B). The calculated odds rate for the High-DEX group was 0.65 (95% CI, 0.54 to 0.78, *P* < 0.001) compared to the Non-DEX group and it was 0.29 (95% CI, 0.22 to 0.39, *P* < 0.001) for the Low-DEX compared to the Non-DEX group. Patients in the High-DEX group showed higher incidence of bradycardia than those in the Non-DEX (20.7% vs. 15.7%, *P* < 0.001) and Low-DEX (20.7% vs. 14.3%, *P* < 0.001) groups. The incidence of hypotension in the High-DEX group was higher than that in the Non-DEX group (31.6% vs. 25.8%, *P* < 0.001). No significant difference was found in bradycardia or hypotension between the Non-DEX and Low-DEX groups (*P* > 0.05). In addition, hospital stay in the Low-DEX group was significantly shorter than that in the High-DEX and Non-DEX group (*P* < 0.001, Table 1).

In the logistic regression analysis, to explore the effect factors for SSD, the model was significant when *P* < 0.001. Preoperative cardiac and pulmonary disease, operation time period, surgery type, and whether the patient received PCIA were identified as significant effect factors (*P* < 0.05, Table 2). Regarding dexmedetomidine use, the odds rates for the Low-DEX and High-DEX groups were 0.32 (95% CI, 0.23 to 0.43, *P* < 0.001) and 0.68 (95% CI, 0.57 to 0.83, *P* < 0.001), respectively, compared with those for the Non-DEX group.

**Table 2:** Potential effect factors on severe sleep disturbance after surgery in an enter logistic regression analysis model.

**Factors**	**Wald *x*^**2**^**	***P*-value**
Sex (Male or female)	2.665	0.100
Age group (≤ 70, 71–75 or >75)	4.612	0.099
BMI group (<18.5, 18.5–25 or >25)	1.617	0.445
ASA class (II or III)	0.659	0.417
Smoking status (yes or no)	1.503	0.220
Hypertension (yes or no)	0.090	0.764
Cardiac disease (yes or no)	6.592	0.010
Diabetes (yes or no)	0.371	0.543
Pulmonary disease (yes or no)	3.991	0.046
Operation time period (Morning or afternoon)	7.603	0.006
Surgery time (<2 or ≥2 h)	0.849	0.357
Surgery type (Abdominal, urological, orthopedic, or other surgery)	21.056	<0.001
Minimally invasive surgery (yes or no)	0.479	0.489
General anesthesia (yes or no)	3.768	0.052
Dexmedetomidine use (Non-DEX, Low-DEX or High-DEX)	59.392	<0.001
Received PCIA (yes or no)	3.920	0.048

*BMI, body max index; ASA, American society of anesthesiologists; PCIA, patient-controlled intravenous analgesia*.

**Figure 4:**
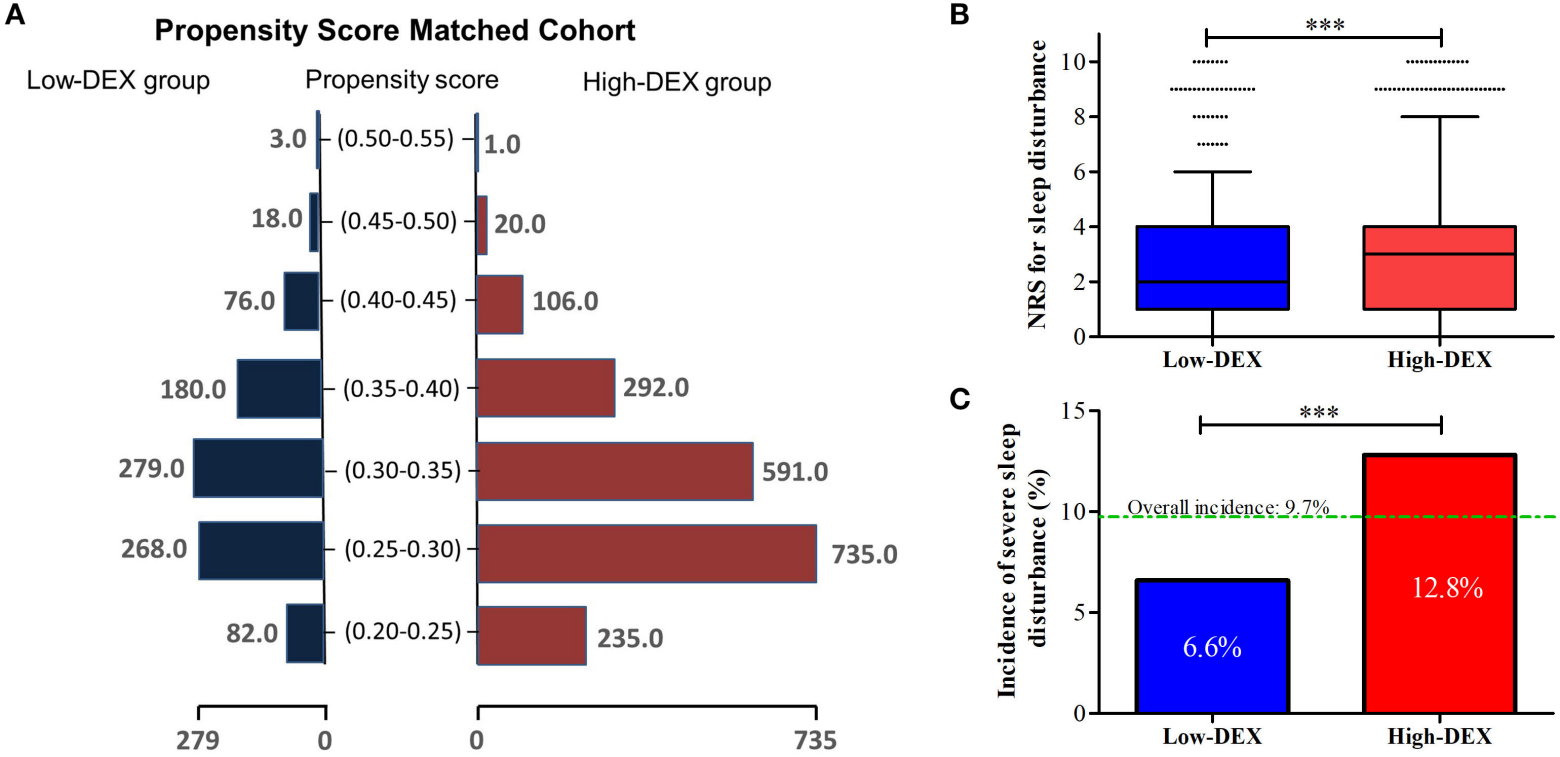
Propensity score-matched analysis for comparisons between Low-DEX and High-DEX group patients. **(A)** Distributions of elderly surgery patients in the Low-DEX and High-DEX groups in propensity score-matching. **(B,C)** NRS for sleep disturbance and the incidence of SSD during the first night after the surgery in Low-DEX (*n* = 906) and High-DEX group (*n* = 906) patients after PSM analysis. ^***^P < 0.001; **(B)** box plot, interquartile range; solid line, median; whiskers, third quartile + 1.5^*^ interquartile range; dots, abnormal value. **(C)** box, percentage.

Then, based on these identified factors in the logistic model and the baseline factors with differences (*P* < 0.05, Table 1) between patients of the Low-DEX and High-DEX groups, we performed a PSM analysis, and it yielded 906 patients in the Low-DEX group matched with 906 patients in the High-DEX group (Figure 4A). The results are shown in Table 3, and no significant difference was found in any basal factor. Distributions of NRS for sleep disturbance in different groups are presented in Figure 4B, the Low-DEX group showed lower NRS (2.7 ± 2.1 vs. 3.1 ± 2.4, *P* < 0.001) than the High-DEX group. The incidence of SSD was lower in the Low-DEX group than in the High-DEX group (6.6% vs. 12.8%, *P* < 0.001, Figure 4C). The calculated odds rate for the Low-DEX group was 0.48 (95% CI, 0.35 to 0.67, *P* < 0.001) compared with that for the High-DEX group. In addition, hospital stay in the Low-DEX group was significantly shorter than that in the High-DEX group (*P* < 0.001) and no significant difference was found in the incidence of independent off-bed activity or additional analgesia requirement (Table 3).

**Table 3:** Comparisons of the clinical characteristics and postoperative outcomes between Low-DEX and High-DEX patient groups after PSM analysis.

	**Low-DEX (*n* = 906)**	**High-DEX (*n* = 906)**	***P*-value**
Age; year	71.9 ± 5.8	71.8 ± 5.6	0.772
Age group; *n* (%)			
≤ 70	433 (47.8%)	430 (47.5%)	0.757
71–75	256 (28.2%)	254 (28.0%)	
>75	217 (24.0%)	222 (24.5%)	
Male; *n* (%)	535 (59.1%)	530 (58.5%)	0.849
BMI; kg/m^2^	23.8 ± 3.6	23.6 ± 3.5	0.184
BMI group; *n* (%)			0.757
<18.5	58 (47.8%)	51 (47.5%)	
18.5–25	540 (28.2%)	550 (28.0%)	
>25	308 (24.0%)	305 (24.5%)	
ASA; *n* (%)			0.524
Class II	332 (36.6%)	318 (35.1%)	
Class III	574 (63.4%)	588 (64.9%)	
Smoking status; *n* (%)	106 (11.7%)	85 (9.4%)	0.126
Hypertension; *n* (%)	341 (37.6%)	300 (33.1%)	0.055
Cardiac disease; *n* (%)	142 (15.7%)	144 (15.9%)	0.949
Diabetes; *n* (%)	123 (13.6%)	138 (15.2%)	0.349
Pulmonary disease; *n* (%)	284 (31.9%)	289 (31.3%)	0.840
Operation at morning; *n* (%)	406 (44.8%)	424 (46.8%)	0.429
Surgery duration; h	2.3 ± 1.3	2.3 ± 1.4	0.954
Major surgery (≥2 h); *n* (%)	452 (49.9%)	455 (50.2%)	0.925
Surgery type; *n* (%)			0.980
Abdominal surgery	176 (19.4%)	182 (20.1%)	
Urological surgery	256 (28.3%)	252 (27.8%)	
Orthopedic surgery	295 (32.6%)	297 (32.8%)	
Other type surgery	179 (19.8%)	175 (19.3%)	
Minimally invasive surgery; *n* (%)	310 (34.2%)	328 (36.2)	0.403
General anesthesia; *n* (%)	595 (65.7%)	593 (65.5%)	0.961
Received PCIA; *n* (%)	383 (42.3%)	386 (42.6%)	0.924
Pain NRS at 24 h after surgery	1.3 ± 1.3	1.3 ± 1.4	0.975
Pain NRS at 48 h after surgery	1.5 ± 1.2	1.6 ± 1.2	0.088
Additional analgesia requirement during 48 h after surgery; *n* (%)	92 (10.2%)	104 (11.5%)	0.405
Sleep quality NRS	2.7 ± 2.1	3.1 ± 2.4	<0.001
Severe sleep disturbance; *n* (%)	60 (6.6%)	116 (12.8%)	<0.001
Independently off-bed activity at 48 h after surgery; *n* (%)	153 (16.9%)	158 (17.4%)	0.803
Hospital stay; day	6.0 ± 2.5	6.7 ± 2.6	<0.001

*BMI, body max index; ASA, American society of anesthesiologists; NRS, number rating scale; PCIA, patient-controlled intravenous analgesia*.

Subgroup analysis with step-wise logistic regression analysis in the patients undergoing general and non-general anesthesia were, respectively, performed. As shown in Table 4, in the general anesthesia population, intraoperative dexmedetomidine dose (*P* < 0.001) and surgery type (orthopedic surgery, *P* < 0.001) were identified as effect factors for SSD, while in the non-general anesthesia population, intraoperative dexmedetomidine dose (*P* = 0.026) and receiving PCIA (*P* = 0.002) were identified. The odds rate in the Low-DEX group in the general anesthesia population compared with that in the High-DEX group was 0.39 (95% CI, 0.27 to 0.57, *P* < 0.001) and that in the non-general anesthesia population was 0.59 (95% CI, 0.37 to 0.94, *P* < 0.001).

**Table 4:** Risk factors for severe sleep disturbance among patients who received dexmedetomidine therapy under general anesthesia and non-general anesthesia in a stepwise logistic regression analysis model.

**Population**	**Factors with statistical significance**	***P*-value**	**OR**	**95% CI**	**Model *P*-value**
General anesthesia (*n* = 2,050)					<0.001
	Intraoperative dexmedetomidine dose (Ref. High-DEX)	<0.001	0.39	0.27–0.57	
	Surgery type (Ref. other type surgery)				
	Abdominal surgery	0.369	0.38	0.56–1.24	
	Orthopedic surgery	<0.001	1.93	1.37–2.72	
	Urological surgery	0.527	0.85	0.52–1.40	
Non-general anesthesia (*n* = 925)					0.022
	Intraoperative dexmedetomidine dose (Ref. High-DEX)	0.026	0.59	0.37–0.94	
	Receiving PCIA (Ref. yes)	0.002	2.22	1.34–3.69	

*OR, odds rate; CI, confidence interval; PCIA, patient-controlled intravenous analgesia; Ref., reference*.

## Discussion

Based on the current study, the results showed that for elderly surgery patients, both a low dose (0.1–0.2 μg/kg/h) and a high dose (>0.2 μg/kg/h) of intraoperative dexmedetomidine can significantly decrease the incidence of first night SSD after surgery compared to patients who did not use dexmedetomidine during the surgery. Interestingly, compared to high-dose dexmedetomidine, a low dose has far better improvement effects for postoperative sleep quality. This finding was further validated in the PSM and subgroup analyses.

In the study, intraoperative dexmedetomidine use was found to be associated with better sleep quality and lower incidence of SSD. Exploring the use of dexmedetomidine to improve sleep quality in elderly patients has been reported through polysomnography recordings in the intensive care unit (12). Although in this study dexmedetomidine was used after surgery, it proved that dexmedetomidine has the potential to improve the first night sleep quality of postoperative surgical patients by changing the sleep structure. In a recent study, intraoperative dexmedetomidine was further explored in respect to its effect on postoperative sleep through polysomnography recording (14). This study demonstrated that using dexmedetomidine during a daytime operation can better improve the first night sleep quality. However, a control group without intraoperative dexmedetomidine use was absent in this study. Thus, it remains unclear whether intraoperative dexmedetomidine use has the effect of changing the sleep structure. Currently, no large sample size study has explored the sleep improvement effect of intraoperative dexmedetomidine. In this retrospective study, more than 4,000 elderly surgery patients were included and compared to Non-DEX patients; both patients in the Low-DEX and High-DEX groups showed better sleep after surgery. Thus, the current finding provides powerful evidence for the improvement of postoperative sleep quality through intraoperative dexmedetomidine use.

Furthermore, the current study found that low-dose (0.1–0.2 μg/kg/h) dexmedetomidine had far better improvement effects for postoperative sleep quality compared to the high dose (>0.2 μg/kg/h). In the retrospective cohort, compared to Non-DEX group patients, dexmedetomidine use could decrease the risk of SSD to 2/3 in the High-DEX group and 1/3 in the Low-DEX group. Then, we used PSM analysis to exclude the potential bias induced by basal confound factors. The results further validated that the NRS for sleep disturbance and incidence of SSD in the Low-DEX group were lower than those in the High-DEX group. Moreover, the odds rate reached 0.48 (95% CI, 0.35 to 0.67), indicating that low-dose dexmedetomidine can decrease about half of the risk for postoperative SSD compared to the high dose. Therefore, based on the current results, we concluded that intraoperative low-dose (0.1–0.2 μg/kg/h) dexmedetomidine might be optimal for improving the sleep quality after surgery for elderly patients.

The reason for the different effects of sleep improvement between the High-DEX and Low-DEX groups remains unknown. A U-shaped benefit curve may exist in intraoperative dexmedetomidine use. Currently, many studies have confirmed that dexmedetomidine use in intensive care units can effectively decrease the incidence of delirium (20, 21). However, several randomized clinical trials found that the reduction in delirium previously demonstrated in intensive care unit studies was not observed when dexmedetomidine was intraoperatively infused during the surgery (22–24). As we know, sleep disorder is an important component for diagnosing delirium (25, 26), as previous studies have reported that poor sleep after surgery is associated with a higher incidence of delirium in elderly patients (27, 28). Thus, these studies seemed not to support the intraoperative use of dexmedetomidine to improve postoperative sleep quality. We found that in the above three studies, the intraoperative dexmedetomidine dose was fixed at 0.4 or 0.5 μg/kg/h. In contrast, one recent randomized study with a small sample size demonstrated that intraoperative continuous infusions of dexmedetomidine (0.1 μg/kg/h) can reduce the incidence and intensity of delirium and also improve the first night sleep quality in elderly patients (13). Also, intraoperative low-dose infusions of dexmedetomidine (0.2 μg/kg/h) was found to reduce the emergence of agitation after strabismus surgeries (29). Thus, the current results further supported the fact that intraoperative low-dose dexmedetomidine might have the best effect on improving sleep quality after surgery.

Beyond that, regarding the postoperative complications for which dexmedetomidine may be incriminated, we found that High-DEX would increase the risk of bradycardia and hypotension compared to Non-DEX. However, different from High-DEX, Low-DEX did not increase these risks. This might also contribute to the different effects on sleep improvement between High-DEX and Low-DEX. This also indicated that intraoperative use of low-dose dexmedetomidine might be a safe strategy to improve the postoperative sleep quality. The effects of intraoperative dexmedetomidine on postoperative pain treatment should also be noted in the study. Poor sleep after surgery has been demonstrated to be associated with more severe postoperative pain (30, 31). However, in this study, no difference was found in postoperative pain between the Low-DEX and High-DEX groups. This indicated that a better effect of intraoperative low-dose dexmedetomidine on sleep improvement might depend on its direct effect on sleep. However, the results showed that the mean pain NRS (<2) and the incidence of additional pain treatment (about 10%) was relatively low in the current population. Thus, considering the low NRS and incidence, the effect of intraoperative dexmedetomidine on postoperative pain might have been underpowered.

In addition, in the subgroup analysis, we found that in both the general and non-general anesthesia population, low-dose dexmedetomidine had the best sleep improvement effect. At present, most of the trials focused on the general anesthesia population to explore the effect of intraoperative dexmedetomidine on postoperative delirium, emergence agitation, or sleep (11, 12, 22, 23, 32). To the best of our knowledge, the current study was the first to include non-general anesthesia patients to explore the sleep improvement effect of low-dose intraoperative dexmedetomidine use. One study has suggested that intraoperative dexmedetomidine sedation has a greater beneficial effect in reducing agitated behavior in elderly patients undergoing orthopedic surgery with regional anesthesia compared to propofol, but the dose was set at a continuous 0.1–0.5 μg/kg/h (33). Another study regarding patients undergoing vitreoretinal surgery demonstrated that adding low-dose dexmedetomidine (about 0.2 μg/kg/h, used as adjuvant for block) to levobupivacaine in vitreoretinal surgery could provide better sleep quality in the first postoperative night compared with levobupivacaine alone (34). This evidence further supported the current finding that it was also effective using low-dose intraoperative dexmedetomidine to improve the sleep quality after surgery.

There are several limitations that should be considered in the current study. Firstly, because of the retrospective design of the current study, the protocol for the included patients' intraoperative operation and medication was difficult to standardize. Secondly, as some previous studies, subjective sleep quality were evaluated using the NRS (16, 17) and no polysomnography parameter was analyzed in the current study. Thus, the underlying evidence for the improvement effect of low-dose dexmedetomidine during surgery on sleep quality needs to be further explored. In addition, only the first night sleep quality was recorded in the study and the time effect of the intraoperative dexmedetomidine remains unclear. Thirdly, in the hospital, most of cardiac surgery patients were sent to an intensive care unit without tracheal tube extubation after the surgery. The postoperative treatment is markedly different between cardiac surgery patients and non-cardiac surgery patients and we did not include cardiac surgery patients in the study. Thus, further study is needed to explore the effect of intraoperative dexmedetomidine on cardiac surgery patients' postoperative sleep quality. However, one strength of the current study should be noted. Different from previous studies, this study retrospectively included a relatively large sample size of elderly surgery patients to explore a better pharmacological strategy to intraoperatively prevent postoperative sleep disorder in a real-world background. This clinical finding was consistently validated in group comparisons, PSM analysis, and sub-group analysis.

## Conclusion

In summary, for elderly surgery patients, intraoperative dexmedetomidine use can significantly improve the first night sleep quality after surgery. Low-dose (0.1–0.2 μg/kg/h) intraoperative dexmedetomidine can provide a better improvement effect on sleep quality and thus 0.1–0.2 μg/kg/h should be recommended during surgery when considering postoperative sleep improvement.

## Data Availability Statement

The datasets analyzed in this article are not publicly available due to patient confidentiality issues, and to allow analysis of other outcomes included in the data. Requests to access the datasets should be directed to TZ, 739501155@qq.com.

## Ethics Statement

The studies involving human participants were reviewed and approved by Institutional Review Boards of The Second Affiliated Hospital, Chongqing Medical University. Written informed consent for participation was not required for this study in accordance with the national legislation and the institutional requirements.

## Author Contributions

JC, YC, XH, XZ, and YT contributed to data collection. JC and YC contributed to data analysis. JC, YC, SW, and TZ contributed to drafting and revising the article. All authors have read and approved the final manuscript.

## Conflict of Interest

The authors declare that the research was conducted in the absence of any commercial or financial relationships that could be construed as a potential conflict of interest.
